# Neurofilament Light Chain as Biomarker for Amyotrophic Lateral Sclerosis and Frontotemporal Dementia

**DOI:** 10.3389/fnins.2021.679199

**Published:** 2021-06-21

**Authors:** Federico Verde, Markus Otto, Vincenzo Silani

**Affiliations:** ^1^Department of Neurology–Stroke Unit and Laboratory of Neuroscience, Istituto Auxologico Italiano, Istituto di Ricovero e Cura a Carattere Scientifico (IRCCS), Milan, Italy; ^2^Department of Pathophysiology and Transplantation, “Dino Ferrari” Center, Università degli Studi di Milano, Milan, Italy; ^3^Department of Neurology, Ulm University Hospital, Ulm, Germany

**Keywords:** amyotrophic lateral sclerosis, frontotemporal dementia, cerebrospinal fluid, biomarkers, neurofilament light chain

## Abstract

Amyotrophic lateral sclerosis (ALS) and frontotemporal dementia (FTD) are two related currently incurable neurodegenerative diseases. ALS is characterized by degeneration of upper and lower motor neurons causing relentless paralysis of voluntary muscles, whereas in FTD, progressive atrophy of the frontal and temporal lobes of the brain results in deterioration of cognitive functions, language, personality, and behavior. In contrast to Alzheimer’s disease (AD), ALS and FTD still lack a specific neurochemical biomarker reflecting neuropathology *ex vivo*. However, in the past 10 years, considerable progress has been made in the characterization of neurofilament light chain (NFL) as cerebrospinal fluid (CSF) and blood biomarker for both diseases. NFL is a structural component of the axonal cytoskeleton and is released into the CSF as a consequence of axonal damage or degeneration, thus behaving in general as a relatively non-specific marker of neuroaxonal pathology. However, in ALS, the elevation of its CSF levels exceeds that observed in most other neurological diseases, making it useful for the discrimination from mimic conditions and potentially worthy of consideration for introduction into diagnostic criteria. Moreover, NFL correlates with disease progression rate and is negatively associated with survival, thus providing prognostic information. In FTD patients, CSF NFL is elevated compared with healthy individuals and, to a lesser extent, patients with other forms of dementia, but the latter difference is not sufficient to enable a satisfying diagnostic performance at individual patient level. However, also in FTD, CSF NFL correlates with several measures of disease severity. Due to technological progress, NFL can now be quantified also in peripheral blood, where it is present at much lower concentrations compared with CSF, thus allowing less invasive sampling, scalability, and longitudinal measurements. The latter has promoted innovative studies demonstrating longitudinal kinetics of NFL in presymptomatic individuals harboring gene mutations causing ALS and FTD. Especially in ALS, NFL levels are generally stable over time, which, together with their correlation with progression rate, makes NFL an ideal pharmacodynamic biomarker for therapeutic trials. In this review, we illustrate the significance of NFL as biomarker for ALS and FTD and discuss unsolved issues and potential for future developments.

## Introduction

Amyotrophic lateral sclerosis (ALS) and frontotemporal dementia (FTD) are two related neurodegenerative diseases. ALS is the most common motor neuron disease (MND) and affects both upper motor neurons (UMNs) located in the cerebral cortex and lower motor neurons (LMNs) located in the brainstem and spinal cord. Their degeneration results in progressive paralysis of voluntary muscles leading to death from respiratory failure after a median of 3–5 years from symptom onset (Masrori and Van Damme, 2020). The large majority of ALS cases occur sporadically, but 5–10% of ALS patients have a family history, usually with autosomal dominant inheritance, and approximately two thirds of them harbor mutations in one (or sometimes more) of > 20 genes, the most common being the (G_4_C_2_)_n_ hexanucleotide repeat expansion (HRE) in the *C9orf72* gene and mutations in the genes *SOD1*, *TARDBP*, and *FUS* (Mejzini et al., 2019). The diagnosis of ALS is fundamentally clinical and is supported by investigations such as electromyography. There is no effective therapy for ALS, with the only two approved specific drugs, riluzole and edaravone, producing modest beneficial effects in terms of prolongation of survival and slowing of functional deterioration, respectively (Masrori and Van Damme, 2020).

FTD is the second most common form of dementia in people younger than 65 years and is classified into three main variants: the behavioral variant (bvFTD) and the two classic variants of primary progressive aphasia (PPA), i.e., the non-fluent/agrammatic variant (nfvPPA) and the semantic variant (svPPA) (Convery et al., 2019). The third PPA variant, the logopenic one (lvPPA), is usually considered separately because it is most commonly due to underlying AD pathology. While bvFTD is characterized by progressive changes in personality and behavior accompanied by a dysexecutive type of cognitive deterioration, the PPAs show selective deficits of speech production (nfvPPA) or of semantic knowledge (svPPA) resulting in progressive language disturbance and absent or only limited impairment of other cognitive domains. Also for FTD, there is no effective therapy, and therefore, the disease poses a heavy burden on often relatively young patients and caregivers (Convery et al., 2019). The neuropathological substrate of bvFTD, nfvPPA, and svPPA is frontotemporal lobar degeneration (FTLD), which is characterized by progressive atrophy of the frontal and temporal lobes of the brain and is in turn classified into three distinct pathological entities: FTLD-tau (comprising approximately 45% of cases), FTLD-TDP (50%), and the rare FTLD-FUS (5%), characterized by intracellular inclusions of pathologically modified forms of the proteins tau, TDP-43 and FUS, respectively (Mann and Snowden, 2017).

TDP-43 pathology is the fundamental element linking FTD and ALS: indeed, TDP-43 inclusions are the neuropathological substrate of virtually all sporadic and the large majority of genetic ALS cases (Neumann et al., 2006). Accordingly, up to 40–50% of ALS patients show at least subtle cognitive or behavioral alterations of the FTD spectrum upon specific neuropsychological investigation, with up to 10–15% fulfilling diagnostic criteria for FTD itself. The co-occurrence of the two diseases is, indeed, not uncommon, both at the individual level and within families, especially considering that the *C9orf72* HRE can cause not only ALS but also FTLD-TDP or both (Masrori and Van Damme, 2020). The other two main genes causing familial forms of FTD, which overall represent up to a third of all FTD cases, are *GRN* and *MAPT*, associated, respectively, with FTLD-TDP and FTLD-tau (Convery et al., 2019). ALS and FTD due to FTLD-TDP are now most commonly considered as two diseases belonging to the same neuropathological spectrum of TDP-43 proteinopathies (de Boer et al., 2020). A further, far less common, neuropathological link between ALS and FTD is represented by FUS pathology, occurring in rare instances of both diseases; however, notably, whereas cases of ALS with FUS pathology are associated with mutations in the corresponding gene *FUS*, the vast majority of FTLD-FUS cases appear to be sporadic (Mann and Snowden, 2017).

Both for ALS and for FTD, especially in the last years, considerable developments have taken place in the field of neurochemical biomarkers, following the example of the successfully established cerebrospinal fluid (CSF) biomarkers of AD (Blennow et al., 2010). Biomarkers are needed for several aims: to support clinicians in the diagnosis and especially in the differential diagnosis; to enable early diagnosis, thus allowing prompt initiation of disease-modifying treatments or early enrollment of patients in clinical trials; to stratify patients in the trials; to demonstrate target engagement by an experimental treatment; and to measure treatment effects as pharmacodynamic biomarkers. The most investigated biomarkers in ALS and FTD are the neurofilaments and especially the light chain (NFL) (Verde et al., 2019a; Swift et al., 2021). Neurofilaments are structural components of the axonal cytoskeleton and are released from the axon as a consequence of its damage or degeneration. This increases their concentration in the CSF and hence in the blood compared with physiological conditions and constitutes the foundation of their use as biomarkers for neurological diseases.

## Neurofilaments: Physiology, Biological Rationale as Biomarkers, and Measurement

**FIGURE 1:**
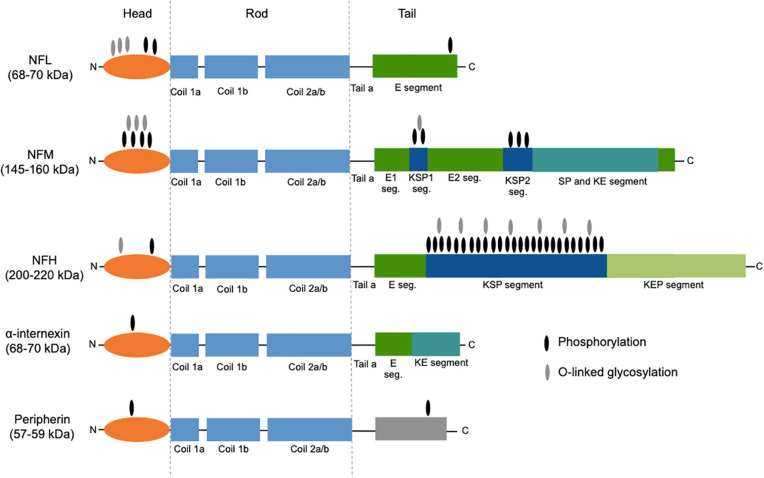
Structure of neurofilament subunits. All neurofilament subunits share a common structure composed of a globular amino-terminal head domain, a central conserved α-helical rod domain (comprising several coiled coils), and a carboxy-terminal tail of variable length. NFM and NFH are characterized by long C-terminal tail domains rich in heavily phosphorylated serine-proline-lysine (KSP) repeats. The two main posttranslational modifications, namely, phosphorylation and O-linked glycosylation, are shown. The figure was created taking Figure 3 from Yuan et al. (2017) as model, with the authors’ permission. NFL, neurofilament light chain; NFM, neurofilament middle chain; NFH, neurofilament heavy chain; E segment (seg.), glutamic acid-rich segment; KSP, lysine-serine-proline; SP, serine-proline; KE, lysine-glutamic acid; KEP, lysine-glutamic acid-proline.

Neurofilaments are a class of intermediate filaments and are a major constituent of the cytoskeletal scaffold of the central (CNS) and peripheral nervous system (PNS) neurons (Yuan et al., 2017). They are most abundant in large myelinated axons; smaller amounts are also found in cell bodies, dendrites, and synapses (Gafson et al., 2020). Neurofilaments are heteropolymers composed of four different subunits: the light, middle, and heavy chains (NFL, NFM, and NFH, respectively), plus α-internexin in the CNS and peripherin in the PNS (Yuan et al., 2017). All neurofilament subunits comprise an N-terminal head domain, a highly conserved central rod domain, and a tail domain of variable length. In addition to this molecular structure, they undergo posttranslational modifications, including phosphorylation, O-linked glycosylation, nitration, and ubiquitination. The molecular weights of the different subunits, predicted based on the genetic sequence, are as follows: 112.5, 102.5, 61.5, 55.4, and 53.7 kDa (NFH, NFM, NFL, alpha-internexin, and peripherin, respectively). Because of the abundance in glutamate residues and of posttranslational modifications, when measured by means of sodium dodecyl sulfate polyacrylamide gel electrophoresis (SDS-PAGE), their molecular weights are slightly higher, namely, 200–220, 145–160, 68–70, 58–66, and 57–59 kDa, respectively (Figure 1). The backbone of neurofilaments is composed of NFL plus α-internexin or peripherin, all three having short tail domains, while NFM and NFH are placed more peripherally, with their long tails projecting radially (Yuan et al., 2017).

**FIGURE 2:**
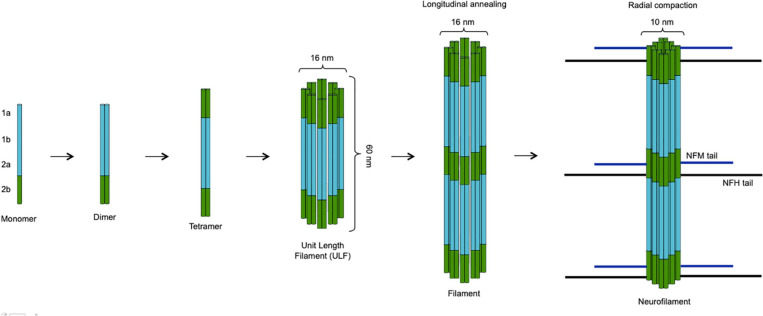
Assembly of neurofilaments. Two neurofilament subunits combine to form a paired coiled-coil dimer, which then undergoes antiparallel association with another dimer giving rise to a non-polar tetramer. The circumferential association of eight tetramers produces a cylindrical unit length filament (ULF) with a diameter of 16 nm. ULFs undergo end-to-end annealing forming elongated filaments, whose radial compaction results in reduction of the diameter to the typical 10-nm size of intermediate filaments. While the backbone of the neurofilament scaffold is constituted by NFL plus α-internexin or peripherin (in the CNS and in the PNS, respectively), NFM and NFH molecules associate more peripherally with their long tails projecting outside and interacting with other cytoskeletal components (microtubules) and organelles (mitochondria). The figure was created taking Figure 4 from Yuan et al. (2017) as model, with the authors’ permission.

The basic assembly of neurofilaments is a paired coiled-coil dimer, which then associates in an antiparallel way with another dimer to form a non-polar tetramer. Eight tetramers associate circumferentially giving rise to a cylindrical structure called unit length filament (ULF). ULFs then undergo end-to-end annealing and radial compaction forming elongated structures with the typical 10-nm diameter of intermediate filaments. These structures have side arms constituted by the long tails of NFH and NFM, which are important for connecting neurofilaments among them as well as with other cytoskeletal components and cellular organelles, e.g., microtubules and mitochondria (Figure 2; Yuan et al., 2017). NFM and NFH contain the most abundant phosphorylation sites, and neurofilament phosphorylation inhibits proteolysis and is therefore important for the structural stability of the whole scaffold (Gafson et al., 2020). Neurofilaments are synthesized in the cell body and are then rapidly but intermittently transported distally along axons, resulting in a net slow anterograde movement, in accordance with the slow turnover of the whole neurofilament network of myelinated axons (Roy et al., 2000; Gafson et al., 2020). At the synaptic level, neurofilaments are more abundant in the postsynaptic compartment, and the synaptic neurofilament pool shows structural as well as biochemical differences from the axonal one (Yuan et al., 2003). The factors regulating neurofilament turnover are not precisely known; degradation involves the ubiquitin–proteasome system and probably also autophagy (Gafson et al., 2020). Neurofilaments have several biological functions: most notably, they are important for the stability of axons, especially of large myelinated ones, and for their radial growth, which determines their fast conduction properties (Barry et al., 2012). They also contribute to maintaining the stability of the mitochondria and the cytoskeletal content of microtubules (Bocquet et al., 2009; Gentil et al., 2015). At the synaptic level, they have a role in maintaining the structure and function of dendritic spines as well as a role in regulating glutamatergic and dopaminergic neurotransmission: as an example, NFL interacts with the cytoplasmic C-terminal domain of the GluN1 subunit of the NMDA glutamate receptor (Yuan et al., 2015; Yuan et al., 2018).

Alterations of neurofilament structure and function could be involved in the pathogenesis of neurodegenerative diseases, with the largest body of evidence pointing toward a possible role in ALS. Neurofilament accumulations are observed in spinal motor neurons in ALS (Corbo and Hays, 1992), and alterations of the genes coding for NFH and peripherin were found in a small number of ALS patients (Figlewicz et al., 1994; Gros-Louis et al., 2004). A mutation of the NFL gene in a genetic mouse model of ALS causes degeneration of spinal motor neurons with accumulation of neurofilaments and atrophy of skeletal muscle (Lee et al., 1994), while genetic deletion of the NFL subunit in the *SOD1*-mutant ALS mouse model slowed disease onset and progression, at the same time reducing selectivity of the disease process toward motor neurons (Williamson et al., 1998). Noteworthy is also the finding that TDP-43 binds the mRNA of NFL and stabilizes it, preventing its degradation (Strong et al., 2007).

**FIGURE 3:**
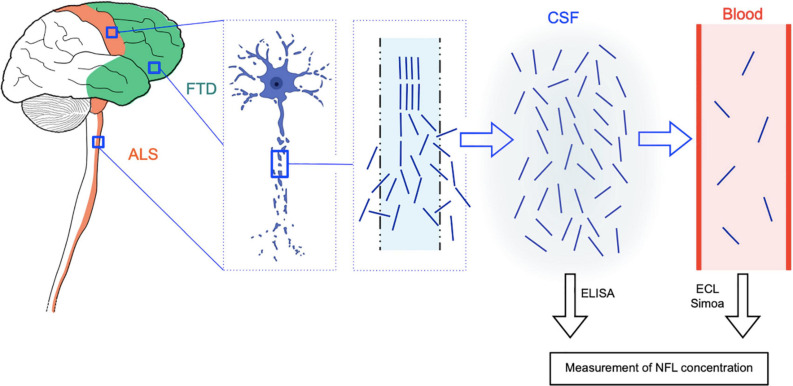
Rationale of NFL as CSF and blood biomarker for ALS and FTD. ALS is characterized by degeneration of upper motor neurons of the motor cortex and of lower motor neurons of the brainstem and spinal cord, while in FTD, the disease process affects neurons of the frontal and temporal cortices. In both diseases, neuroaxonal degeneration causes the release of neurofilament (and in particular NFL) molecules from the axon to the interstitial fluid and hence to the CSF and finally to the blood, where they are present at much lower concentrations (approximate CSF:blood ratio for NFL: 40:1). NFL levels in the CSF can be quantified by means of conventional ELISA assays, while more sensitive techniques are needed for the measurement of the lower blood (serum or plasma) levels, i.e., electrochemiluminescence (ECL) and the ultrasensitive single molecule array (Simoa) technology. The image of the degenerating neuron was taken from BioRender.com (2021).

The role of neurofilaments as biomarkers is thought to be due to their release through the axonal plasma membrane as a consequence of axonal damage or degeneration (Khalil et al., 2018). Following leakage into the extracellular fluid (ISF) and hence into the CSF, neurofilaments penetrate into the blood, where they are usually present with a concentration gradient of about 1:40 relative to the CSF (Gaetani et al., 2019; Figure 3). This is the reason why elevated neurofilament levels in the CSF and in the blood have been described in a variety of neurological conditions characterized by neuroaxonal damage. This is true not only for neurodegenerative diseases but also for other pathophysiological processes, including multiple sclerosis (MS) (Disanto et al., 2017), HIV encephalopathy (Gisslen et al., 2016), and traumatic brain injury (Shahim et al., 2016). For the above reasons, neurofilaments are rather an unspecific marker of axonal damage/degeneration than a pathology-specific marker as, for example, Aβ or phosphorylated tau for AD (Gaetani et al., 2019). However, in the diagnostic field, they can be useful in discriminating between conditions characterized by a higher vs. a lower amount or rate of degeneration of large myelinated axons. This is the case of ALS, in which NFL elevations exceed those observed in most other neurological diseases (Bridel et al., 2019). Moreover, in the same wide range of neurological conditions, neurofilaments can have prognostic significance: as an example, in MS, baseline CSF and serum NFL levels predict longitudinal functional disability (Disanto et al., 2017).

Neurofilament levels in the CSF can be measured by means of traditional sandwich enzyme-linked immunosorbent assays (ELISAs) (Petzold et al., 2010). A major technological advance has been represented by the introduction of electrochemiluminescence (ECL) assays, enabling the measurement of neurofilaments also in the blood of patients with neurological diseases (Gaiottino et al., 2013). Finally, the introduction of the ultrasensitive single molecule array (Simoa) technology, based on simultaneous digital counting of single capture microbeads, has allowed precise quantification also of the low concentrations of neurofilaments in the blood (serum or plasma) of healthy individuals (Rissin et al., 2010; Gisslen et al., 2016). This has paved the way for an expansion of the field of biomarkers of neurodegenerative diseases from the CSF only toward peripheral blood, with considerable advantages in terms of reduced invasiveness, scalability, and opportunities of longitudinal evaluations. Although current important limitations to a large-scale application of neurofilament measurement with the Simoa technology are represented by its presently limited diffusion outside major research centers and relatively high costs, it can be envisioned that this technology as well as similar ultrasensitive ones will undergo increasing diffusion in the next few years. While in ALS the phosphorylated neurofilament heavy chain (pNFH) has been studied as CSF biomarker to a similar extent as NFL, in the field of FTD and other dementias, NFL has been more widely investigated than pNFH (Verde et al., 2019a; Swift et al., 2021). Moreover, pertaining to measurements in peripheral blood, a larger body of evidence has been produced for NFL, not least because of technical reasons regarding measurement, including the earlier availability of ultrasensitive assays for detecting this neurofilament subunit (Verde et al., 2019b).

## NFL as Biomarker for Amyotrophic Lateral Sclerosis

### NFL as ALS Diagnostic Biomarker

ALS is characterized by a relatively rapid degeneration of motor neurons, and these cells, given their large myelinated axons of considerable length, contain a great amount of neurofilaments. These are the main reasons why ALS shows the most massive elevation of NFL concentrations in the CSF among the commonest neurodegenerative diseases (Olsson et al., 2019). In comparison with neurologically healthy controls, CSF NFL levels are more than sevenfold increased in ALS (Skillbäck et al., 2017; Bridel et al., 2019). Rosengren et al. (1996) were the first to show the potential of NFL as biomarker for ALS: in 1996, they developed a new ELISA assay for NFL and found that the protein was present at higher concentrations in the CSF of 12 ALS patients compared with 34 neurologically healthy individuals. Several years later, increased NFL levels in the CSF of ALS patients were reported also in comparison with other neurological, including neurodegenerative, diseases (Zetterberg et al., 2007; Tortelli et al., 2012).

One of the main investigations on NFL (and also on pNFH) as biomarker of ALS is the large German study by Steinacker et al. (2016) (Table 1). They measured NFL and pNFH levels in the CSF of 253 MND patients, 85 patients presenting with MND mimics (i.e., diseases clinically mimicking MND), and 117 patients with other neurological diseases. The levels of the two neurofilaments were strongly correlated, as confirmed in other cohorts (Rossi et al., 2018). Both neurofilaments showed higher levels in ALS compared with every other diagnostic category. For CSF NFL, this enabled discrimination of MND cases from MND mimics with an area under the ROC curve (AUC) of 0.866 and from all non-MND patients with an AUC of 0.851. In particular, at a cutoff of 2,200 pg/ml, CSF NFL distinguished between MND and MND mimics with 77% sensitivity and 88% specificity. The diagnostic performance of pNFH was similar (Steinacker et al., 2016). Poesen et al. (2017) conducted an analogous study on CSF neurofilaments in a comparably large cohort. Notably, they found that CSF NFL was less specific than pNFH in discriminating between ALS and neurological disease controls, because patients with FTD and some of the patients with inflammatory radiculoneuropathies [i.e., chronic inflammatory demyelinating polyneuropathy (CIDP) and Guillain–Barré syndrome (GBS)] showed NFL elevations in the range of ALS patients. Also in the differentiation between ALS and ALS mimics, NFL performed slightly but significantly less well than pNFH, with an AUC of 0.863 corresponding to a sensitivity of 78.2% and a specificity of 63% (Poesen et al., 2017). Other studies on CSF NFL reported AUCs as high as 0.922 for the discrimination between ALS and ALS mimics (Abu-Rumeileh et al., 2020).

**TABLE 1:** Representative studies on NFL as diagnostic biomarker for ALS.

Study (authors and year)	ALS patients	Controls	Biological fluid	Type of assay	NFL levels in ALS patients (pg/ml; median, range)	NFL levels in controls (pg/ml; median, range)	Cutoff for discrimination between ALS and controls (pg/ml)	AUC (95% CI)	Sensitivity and specificity (95% CI)
Steinacker et al. (2016)	253 MND patients (222 ALS, 11 PLS, 20 familial/genetic ALS)	85 MND mimic patients, 28 AD patients, 26 patients with Parkinsonisms, 33 patients with polyneuropathies, 30 patients with facial palsy	CSF	ELISA	MND: 5,068 (100–38,350) ALS: 4,990 (100–38,350) PLS: 3,750 (100–26,650) Genetic/familial ALS: 6,452 (785–22,040)	MND mimics: 865 (168–10,000) AD: 1,510 (625–9,507) Parkinsonian syndromes: 1,455 (439–4,841) Polyneuropathies: 1,034 (277–24,330) Facial palsy: 585 (100–2,676)	2,200	MND vs. MND mimics: 0.866 (0.821–0.911) MND vs. all control groups: 0.851 (0.813–0.888)	MND vs. MND mimics: Se 77% (71–82%), Sp 88% (79–94%) MND vs. all control groups: Se 77% (71–82%), Sp 85% (79–90%)
Poesen et al. (2017)	220	50 ALS mimic patients 316 neurological disease controls	CSF	ELISA	9,427 (370–108,909)	ALS mimic patients: 1,407 (613–36,597) Neurological disease controls: 1,790 (262–53,677)	ALS vs. ALS mimics: 2,453 ALS vs. disease controls: 3,819	ALS vs. ALS mimics: 0.863 (0.808–0.908) ALS vs. disease controls: 0.809 (0.763–0.849)	ALS vs. ALS mimics: Se 85.4% (78.8–90.6%), Sp 78.0% (64.0–88.5%) ALS vs. disease controls: Se 78.8% (71.4–85.0%), Sp 72.7% (66.0–78.8%)
Feneberg et al. (2018)	48 ALS patients sampled ≤ 6 months after symptom onset (“early ALS”) (CSF: 48; serum: 40) 128 ALS patients sampled > 6 months after symptom onset (“late ALS”) (CSF: 128; serum: 112)	65 patients with ONDs (CSF: 65; serum: 48) 27 patients with MND mimics (CSF: 27; serum: 21)	CSF, serum	ELISA (CSF), Simoa (serum)	Early ALS, CSF: 6,802 (1,053–25,650) Early ALS, serum: 255 (51–879) Late ALS, CSF: 5,266 (985–24,240) Late ALS, serum: 196 (24–4,235)	ONDs, CSF NFL: range 152–4,874 ONDs, serum NFL: range 9–427 MND mimics, CSF NFL: range 219–3,390 MND mimics, serum NFL: range 15–95	CSF NFL, early ALS vs. ONDs: 2,300 CSF NFL, early ALS vs. MND mimics: 2,183 Serum NFL, early ALS vs. ONDs: 128 Serum NFL, early ALS vs. MND mimics: 97 CSF NFL, late ALS vs. ONDs: 2,146 CSF NFL, late ALS vs. MND mimics: 2,089 Serum NFL, late ALS vs. ONDs: 116 Serum NFL, late ALS vs. MND mimics: 95	CSF NFL, early ALS vs. ONDs: 0.95 (0.91–0.99) CSF NFL, early ALS vs. MND mimics: 0.94 (0.94–1) Serum NFL, early ALS vs. ONDs: 0.92 (0.85–0.99) Serum NFL, early ALS vs. MND mimics: 0.99 (0.97–1) CSF NFL, late ALS vs. ONDs: 0.93 (0.9–0.96) CSF NFL, late ALS vs. MND mimics: 0.96 (0.93–0.99) Serum NFL, late ALS vs. ONDs: 0.9 (0.83–0.97) Serum NFL, late ALS vs. MND mimics: 0.97 (0.94–1)	CSF NFL, early ALS vs. ONDs: Se 94% (83–99%), Sp 86% (75–93%) CSF NFL, early ALS vs. MND mimics: Se 89% (71–98%), Sp 94% (83–99%) Serum NFL, early ALS vs. ONDs: Se 88% (73–96%), Sp 92% (80–94%) Serum NFL, early ALS vs. MND mimics: Se 100% (84–100%), Sp 90% (76–97%) CSF NFL, late ALS vs. ONDs: Se 89% (82–93%), Sp 84% (73–92%) CSF NFL, late ALS vs. MND mimics: Se 89% (71–98%), Sp 89% (81–93%) Serum NFL, late ALS vs. ONDs: Se 79% (70–86%), Sp 92% (80–98%) Serum NFL, late ALS vs. MND mimics: Se 100% (84–100%), Sp 84% (76–90%)
Gille et al. (2019)	149	19 ALS mimic patients 82 disease controls (48 GBS, 20 CIDP, 14 HSP)	Serum	ECL	179 (0.3–1,141)	ALS mimics: 29 (6–1,053) Disease controls: GBS: 123 (22–9,045); CIDP: 101 (29–2,863); HSP: 37 (8–639)	ALS vs. ALS mimics: 93 ALS vs. (GBS + CIDP): 139 ALS vs. HSP: 55	ALS vs. ALS mimics: 0.85 (0.79–0.90) ALS vs. (GBS + CIDP): 0.58 (0.51–0.64) ALS vs. HSP: 0.84 (0.78–0.90)	ALS vs. ALS mimics: Se 79.2% (71.8–85.4%), Sp 84.2% (60.4–96.6%) ALS vs. (GBS + CIDP): Sp 63.2% (50.7–74.6%) ALS vs. HSP: Se 89.3% (83.1–93.7%), Sp 78.6% (49.2–95.3%)
Verde et al. (2019b)	124	50 patients without neurodegenerative diseases (non-neurodegenerative controls), 44 patients with conditions in the differential diagnosis of ALS (disease controls), 20 FTD patients, 20 AD patients, 19 PD patients, 6 CJD patients	Serum	Simoa	125 (14.6–908)	Non-neurodegenerative controls: 16.2 (5.4–79.9) Disease controls: 27.3 (0.7–210) FTD: bvFTD: 46.5 (19.4–103); nfvPPA: 49.6 (28.2–124); svPPA: 53.9 (45.3–62.5); PPA, unspecified: 76.3 (51.6–101) AD: 38.6 (21.6–240) PD: 27.5 (7.7–81.5) CJD: 162.5 (121–288)	ALS vs. non-neurodegenerative controls: 49 ALS vs. disease controls: 62 ALS vs. all non-ALS categories: 62	ALS vs. non-neurodegenerative controls: 0.971 (0.950–0.991) ALS vs. disease controls: 0.873 (0.810–0.935) ALS vs. all non-ALS categories: 0.887 (0.849–0.926)	ALS vs. non-neurodegenerative controls: Se 89.5% (82.7–94.3%), Sp 92% (80.8–97.8%) ALS vs. disease controls: Se 85.5% (78.0–91.2%), Sp 77.3% (62.2–88.5%) ALS vs. all non-ALS categories: Se 85.5% (78.0–91.2%), Sp 81.8% (74.9–87.4%)

*AD, Alzheimer’s disease; ALS, amyotrophic lateral sclerosis; AUC, area under the ROC (receiver operating characteristic) curve; CJD, Creutzfeldt–Jakob disease; CI, confidence interval; CIDP, chronic inflammatory demyelinating polyneuropathy; CSF, cerebrospinal fluid; ECL, electrochemiluminescence; ELISA, enzyme-linked immunosorbent assay; FTD, frontotemporal dementia; GBS, Guillain–Barré syndrome; HSP, hereditary spastic paraparesis; MND, motor neuron disease; NFL, neurofilament light chain; nfvPPA, non-fluent variant of primary progressive aphasia; ONDs, other neurological diseases; PD, Parkinson’s disease; PLS, primary lateral sclerosis; PPA, primary progressive aphasia; Se, sensitivity; Simoa, single molecule array; Sp, specificity; svPPA, semantic variant of primary progressive aphasia.*

Feneberg et al. (2018) investigated the diagnostic performance of CSF NFL for ALS patients who are evaluated early in the disease course, i.e., within 6 months from symptom onset: also these patients had higher CSF NFL levels than the other categories, including MND mimics, resulting in good discrimination of ALS from MND mimics with AUC 0.94, sensitivity 89%, and specificity 94%. This supports a role of NFL measurement as an aid to the early diagnosis of ALS, which is fundamental for timely initiation of disease-modifying treatments and for enrollment of patients in clinical trials.

A meta-analysis published in 2019 and examining 11 studies conducted on a total of 1,239 patients with ALS and 806 patients with ALS mimic diseases confirmed the significant difference in CSF NFL levels between the two conditions. This corresponded to a ratio of means of 3.35 in NFL levels between ALS and mimics (Forgrave et al., 2019).

Gaiottino et al. (2013) were the first to measure NFL concentrations in the blood, namely, in the serum, of ALS patients using an ECL assay. ALS patients had higher NFL levels compared with healthy controls and patients with neurological complaints but no evidence of CNS pathology; serum NFL enabled discrimination between ALS and healthy controls with a sensitivity of 91.3% and a specificity of 91%. Importantly, CSF and serum levels of NFL were strongly correlated in ALS (*r* = 0.70), with a mean ratio of concentrations between CSF and serum of 57.8 (Gaiottino et al., 2013). The high correlation between CSF and serum levels of NFL has been subsequently confirmed by several other investigations (Steinacker et al., 2017a; Benatar et al., 2018). The same ECL assay was used in another study to demonstrate increased NFL levels also in the plasma of ALS patients compared with healthy controls, resulting in an AUC of 0.869 (Lu et al., 2015).

Gille et al. (2019) measured, by means of ECL as well, serum NFL in a cohort of patients with ALS (*n* = 149) and patients with ALS mimics and other neurological diseases. Given serum NFL levels on average sixfold higher in ALS compared with ALS mimics, the biomarker enabled differentiation between the two conditions with an AUC of 0.85 (best cutoff: 93 pg/ml). Notably, ALS had significantly higher serum NFL levels compared with the UMN diseases primary lateral sclerosis (PLS) and hereditary spastic paraparesis (HSP), while the differences between ALS and the LMN disease progressive muscular atrophy (PMA), CIDP, and GBS did not reach statistical significance. The performance in discriminating between ALS and PLS was good with an AUC of 0.89, corresponding to a sensitivity of 80.5% and a specificity of 90.9% (Gille et al., 2019).

We and others conducted a similar study investigating serum NFL in ALS patients (*n* = 124), patients with other neurodegenerative diseases, patients with conditions in the differential diagnosis of ALS, and neurological patients without neurodegenerative diseases. Serum NFL levels, measured with the Simoa technology, were significantly higher in ALS compared with each other category with the notable exception of Creutzfeldt–Jakob disease (CJD). In the discrimination between ALS and conditions in its differential diagnosis, serum NFL showed an AUC of 0.873 (best cutoff: 62 pg/ml) (Verde et al., 2019b). Importantly, similar to what has been described for CSF NFL, also serum NFL was shown to be already elevated in ALS patients presenting within 6 months from symptom onset, enabling excellent discrimination from MND mimics with AUC of 0.99 (Feneberg et al., 2018).

The already mentioned meta-analysis of 2019 examined four studies on serum NFL comprising 458 ALS cases and 181 cases of ALS mimics and confirmed significantly higher levels in the former category, with a ratio of means of 8.15 (Forgrave et al., 2019).

### Relationship With Demographic and Clinical Characteristics

Nearly all investigations on CSF NFL in ALS agree that the levels of the biomarker do not differ between male and female patients (Gaiani et al., 2017; Skillbäck et al., 2017; Rossi et al., 2018). However, some studies reported higher blood NFL concentrations in female ALS patients: in one cohort, the finding could be explained by females suffering from more advanced disease (and being on average older than male patients), but in general, it warrants further investigation (Lu et al., 2015; Benatar et al., 2020; De Schaepdryver et al., 2020). Most studies did not find an association between CSF and serum NFL levels and age of ALS patients, whereas in healthy controls and in other neurological diseases, the biomarker is correlated with age (Zetterberg et al., 2007; Rossi et al., 2018; Verde et al., 2019b). This is probably due to the massive elevation of NFL concentrations resulting from axonal degeneration in ALS, which largely exceeds the mildly increased rate of axonal loss occurring in normal aging or the moderately increased rate characterizing slower disease processes such as those observed in other neurodegenerative diseases. A meta-analysis of 2019 confirmed an increase of 3.3% per year of age in CSF NFL levels in healthy controls but not in ALS (Bridel et al., 2019). However, some studies reported a weak correlation between CSF or serum NFL levels and age also in ALS (Gaiottino et al., 2013; Benatar et al., 2020). Although a recent study suggested an association between higher serum NFL levels and bulbar onset in ALS (Benatar et al., 2020), according to most other investigations, CSF and blood concentrations of NFL are not influenced by site of onset (Poesen et al., 2017; Rossi et al., 2018; Verde et al., 2019b).

The relationship of NFL with the differential involvement of UMNs vs. LMNs and with the anatomical extent of disease represents a more complex issue. The early investigation of Rosengren et al. (1996) already reported higher CSF NFL levels in ALS patients showing signs of UMN in comparison with those with LMN signs only. Another study confirmed higher concentrations in patients with typical ALS, ALS with predominant UMN signs (UMN-ALS), and progressive bulbar palsy (PBP) compared with the LMN-predominant variants flail arm and flail leg syndromes and the LMN-only variant PMA (Gaiani et al., 2017). In partial agreement with this, both CSF and serum NFL levels were reported to be higher in ALS patients with clinical UMN and clinical/electromyographic LMN signs in two or three body regions compared with patients with UMN and LMN signs in only one region (Poesen et al., 2017; Gille et al., 2019). Gille et al. (2019) showed that, at least for serum NFL, this effect seemed to depend on UMN involvement, with a significant difference between patients with UMN signs in one vs. three regions and no difference between patients with LMN signs in one vs. two or three regions. Other studies reported a correlation of CSF or serum NFL levels with clinical scores of UMN burden, such as the Penn score, in parallel with a negative correlation with fractional anisotropy (FA) and a positive correlation with radial diffusivity (RD) of the corticospinal tracts (CSTs) in diffusion tensor imaging (DTI) studies, reflecting CST, and therefore UMN, degeneration (Menke et al., 2015; Schreiber et al., 2018). However, not all investigations confirm the association of NFL levels with DTI measures of CST integrity (Steinacker et al., 2016). Contrary to the abovementioned findings, another study on CSF NFL in ALS reported no association with anatomical extent of clinical UMN involvement, a borderline association with UMN + LMN involvement, and an association with the number of regions with electromyographic evidence of LMN degeneration; however, no correlation was found between CSF NFL levels and an EMG-based denervation score (Abu-Rumeileh et al., 2020). In general, the associations between NFL levels and anatomo-clinical features of ALS described above were reported by single or limited numbers of investigations and are therefore controversial; it is likely that the inconsistent results between the different studies are due not only to inherent variability between the relative patient cohorts but also to differences in the methods of measurement of the variables involved.

Pertaining to anatomical extent of motor degeneration, neither CSF nor serum NFL levels differ among patients belonging to the different diagnostic categories of the El Escorial criteria (Feneberg et al., 2018), and serum NFL levels are not influenced by neuroimaging-based disease stages as defined by DTI measures of degeneration of cerebral white matter tracts reflecting the neuropathological staging of ALS (Brettschneider et al., 2013; Verde et al., 2019b). No significant or consistent associations were found between CSF or blood (mostly serum) NFL levels and other clinical data in ALS, including measures of muscle strength based on the scoring system of the Medical Research Council (MRC) (Tortelli et al., 2012), cognitive performance as reflected by a global cognitive *z* score or by the score on the Edinburgh Cognitive and Behavioral ALS Screen (ECAS) (Steinacker et al., 2017a; Illán-Gala et al., 2018), or the albumin quotient in basic biochemical analysis of the CSF, reflecting the function of the blood–CSF barrier (Tortelli et al., 2012; Steinacker et al., 2016). A study reported a weak negative correlation between CSF NFL and forced vital capacity (FVC) at pulmonary function testing, reflecting the strength of respiratory muscles (Poesen et al., 2017). Notably, serum and plasma NFL levels do not differ between patients already taking riluzole and still untreated patients (Lu et al., 2015; Verde et al., 2019b).

### NFL as Prognostic Biomarker

Although some investigations reported a weak correlation of CSF NFL with the score on the revised ALS Functional Rating Scale (ALSFRS-R), reflecting the degree of functional impairment (Tortelli et al., 2012; Steinacker et al., 2016; Schreiber et al., 2018), most studies do not confirm this finding (Illán-Gala et al., 2018; Verde et al., 2019b; Benatar et al., 2020). On the contrary, in the majority of investigated cohorts, including the recent large study by De Schaepdryver et al. (2020) on 383 ALS patients, CSF or serum NFL levels correlate moderately with the disease progression rate as expressed by the number of points lost on the ALSFRS-R score from disease onset divided by the disease duration from onset to sampling expressed in months (Gaiani et al., 2017; Illán-Gala et al., 2018; Verde et al., 2019b; Abu-Rumeileh et al., 2020; De Schaepdryver et al., 2020; Thouvenot et al., 2020; Table 2). In the cohort of Poesen et al. (2017), patients with fast and intermediate progression rates showed significantly higher CSF NFL levels compared with patients with slow disease progression, and the biomarker enabled discrimination between patients with fast and those with slow progression with an AUC of 0.814. The same authors confirmed the findings also for serum NFL, observing significantly higher levels in patients with fast progression compared with those with intermediate or slow progression: this resulted in good discrimination between patients with rapid and those with slow progression with AUC of 0.87 (Gille et al., 2019). The moderate correlation between NFL levels and disease progression rate was confirmed also for plasma and, importantly, when considering the final progression rate, i.e., that measured based on the ALSFRS-R score at the last follow-up visit, which better reflects the entire progression and has stronger prognostic significance due to its longitudinal nature (Lu et al., 2015).

**TABLE 2:** Representative studies on the association of NFL with progression rate and survival in ALS.

Study (authors and year)	ALS patients	Biological fluid	Type of assay	DPR (median, range)	Correlation between NFL and DPR (*r*, 95% CI)	Association between NFL and survival (HR, 95% CI)
Lu et al. (2015)	London cohort (plasma): 103 Oxford cohort (serum/CSF): serum: 64; paired CSF: 38	Plasma, serum, CSF	ECL	n.p.	London cohort, correlation between plasma NFL and DPR at baseline: *r* = 0.47 Oxford cohort, correlation between serum NFL and DPR at baseline: *r* = 0.51 London cohort, correlation between plasma NFL and DPR at last visit: *r* = 0.48 Oxford cohort, correlation between serum NFL and DPR at last visit: *r* = 0.53	London cohort (plasma NFL): mid tertile vs. low tertile: HR = 1.91 (0.86–4.23) London cohort (plasma NFL): high tertile vs. low tertile: HR = 3.78 (1.68–8.50) Oxford cohort (serum NFL): mid tertile vs. low tertile: HR = 2.68 (0.87–8.27) Oxford cohort (serum NFL): high tertile vs. low tertile: HR = 6.05 (1.68–21.87) Oxford cohort (CSF NFL): mid tertile vs. low tertile: HR = 3.64 (0.77–17.25) Oxford cohort (CSF NFL): high tertile vs. low tertile: HR = 31.82 (3.75–269.71)
Steinacker et al. (2016)	253 MND patients (222 ALS, 11 PLS, 20 familial/genetic ALS)	CSF	ELISA	n.p.	*r* = 0.3264 (0.2023-0.4402)	Significant differences between survival curves of patients with CSF NFL ≤ median, between median and 75th percentile, and ≥ 75th percentile
Steinacker et al. (2017a)	125	Serum	ECL	0.48 (0.26–0.75)	*r* = 0.291 (0.1113–0.4515)	Comparison between survival curves of the three tertiles: chi-square = 11.54
Gille et al. (2019)	149	Serum	ECL	0.672 (0.058–5.00)	*r* = 0.51 AUC to discriminate between fast and slow progressors (i.e., low and high tertiles of DPR): 0.87 (95% CI, 0.76-0.94) at a cutoff of 159 pg/ml	Mid vs. low NFL tertile: HR = 4.47 (1.08–18.63) High vs. low NFL tertile: HR = 5.34 (1.39–20.56)
Verde et al. (2019b)	124	Serum	Simoa	0.375 (0–6)	*r* = 0.3359 (0.1404-0.5062)	NFL > median vs. NFL ≤ median: HR = 2.392 (1.236–4.63)
Thouvenot et al. (2020)	207	Serum	Simoa	n.p.	*r* = 0.571	NFL ≥ median vs. NFL < median: HR = 4.7 (3.0–7.4)
Abu-Rumeileh et al. (2020)	80	CSF	ELISA	IQR: 0.24–1.15	*r* = 0.391	High vs. low NFL tertile: HR = 3.943 (1.097–14.167)
De Schaepdryver et al. (2020)	383	Serum	ECL	0.67 (0.03–10.13)	*r* = 0.519 (0.437-0.592)	NFL ≥ median vs. NFL < median: HR 2.21 (1.51–3.24)

*ALS, amyotrophic lateral sclerosis; AUC, area under the ROC (receiver operating characteristic) curve; CI, confidence interval; CSF, cerebrospinal fluid; DPR, disease progression rate; ECL, electrochemiluminescence; ELISA, enzyme-linked immunosorbent assay; HR, hazard ratio; IQR, interquartile range; MND, motor neuron disease; NFL, neurofilament light chain; n.p., not provided; PLS, primary lateral sclerosis; Simoa, single molecule array.*

In parallel, the large majority of studies agree that CSF or blood (mostly serum but also plasma) NFL levels have a moderate negative correlation with disease duration at sampling (Lu et al., 2015; Steinacker et al., 2016; Gaiani et al., 2017; Gille et al., 2019; Verde et al., 2019b; Abu-Rumeileh et al., 2020). This is most probably not due to a change in NFL levels along the natural history of the disease, but rather reflects the fact that patients with a more rapid disease course come earlier to medical attention and are thus investigated with a shorter delay from symptom onset, as demonstrated by the association between rapid disease progression rates and short disease durations at sampling observed in the same cohorts (Gille et al., 2019; Verde et al., 2019b). The consistent correlation with disease progression rate, as compared with the inconsistent associations with anatomical burden of disease, represents the most important feature of NFL as CSF or blood biomarker for ALS apart from its diagnostic potential: indeed, it indicates that NFL levels reflect the rate of degeneration of the motor system (determining the release of NFL from the axons of diseased motor neurons) rather than its spatial extent and can therefore be considered as an index of the biological aggressiveness of the disease (Verde et al., 2019b). This fundamental characteristic of NFL in ALS represents the basis for its role as a prognostic biomarker for the disease. This also explains why PLS has lower NFL levels than ALS, given that, although selectively involving UMNs, it generally has a much slower progression (Gille et al., 2019). A potential drawback of the relationship between NFL and disease progression rate is the possibly lower accuracy of NFL in diagnosing slowly progressive ALS forms, which could especially apply to LMN-predominant cases; however, this issue requires further investigations on large and properly selected patient cohorts.

In agreement with its association with disease progression rate, NFL is also associated with survival according to nearly all studies conducted on CSF (Steinacker et al., 2016; Gaiani et al., 2017; Skillbäck et al., 2017; Illán-Gala et al., 2018; Rossi et al., 2018; Abu-Rumeileh et al., 2020), serum (Steinacker et al., 2017a; Gille et al., 2019; Verde et al., 2019b; De Schaepdryver et al., 2020; Thouvenot et al., 2020), and plasma (Lu et al., 2015). Regarding CSF NFL, Abu-Rumeileh et al. (2020) reported a hazard ratio (HR) of 3.943 for patients with NFL concentrations in the highest tertile compared with those with concentrations in the lowest tertile. As for serum NFL, in the large cohort of De Schaepdryver et al. (2020) of 383 ALS patients, those with NFL levels above the median had a HR of 2.21 compared with those with levels below the median. In their elegant work, Thouvenot et al. (2020) demonstrated that in a multivariate analysis of survival on a cohort of 198 patients, only baseline serum NFL, site of onset, and weight loss were independent predictors of survival, with 0.74% increase in the risk of death for every 1 pg/ml increase in baseline serum NFL concentration.

Other prognostic parameters have been associated with NFL by single investigations. Tortelli et al. (2015) reported a moderate negative correlation between CSF NFL levels and the time to generalization, i.e., the time from onset of symptoms in the bulbar region to involvement of spinal regions in patients with bulbar onset or vice versa for patients with spinal onset: patients with NFL levels above the median had a 7.9-fold increased risk of generalization compared with those with lower NFL levels, corresponding to a shortening of 2.8 months of the time to generalization for every 1,000 pg/ml increase of CSF NFL concentration. Finally, baseline CSF NFL was shown to predict longitudinal functional deterioration in ALS patients as assessed by both the ALSFRS-R and the related Milano-Torino Staging (MiToS) system (Gaiani et al., 2017).

### Longitudinal Kinetics of NFL

A particularly relevant issue is represented by the longitudinal evolution of NFL concentrations over the disease course. In this regard, data on CSF are limited because of the need for repeated lumbar punctures, which have been performed only in subcohorts of patients. The largest investigation is that of Skillbäck et al. (2017), who found increased NFL levels at follow-up CSF examinations in 67% of the 69 MND patients undergoing a repeat lumbar puncture. On the contrary, a decrease of NFL at follow-up was reported for the 11 patients of the large MND cohort of Steinacker et al. (2016) undergoing a second CSF sampling. However, in a slightly larger subcohort of ALS patients, Poesen et al. (2017) found increased follow-up CSF NFL levels in a subset of intermediate and fast progressors, while levels were stable in the remaining patients, including those with slow progression. Finally, Lu et al. (2015) reported a slight increase in both slow and fast progressors, with stable levels in intermediate progressors. The same authors investigated longitudinal kinetics of NFL also in the blood, whereby the less invasive sampling procedure enables the study of larger cohorts and therefore more solid conclusions. While in the cohort with plasma samples NFL levels did not change significantly longitudinally, in the serum cohort (overlapping with the CSF cohort mentioned above), a slight increase of NFL was observed in fast progressors, with stable levels in slow and intermediate progressors (Lu et al., 2015). Other studies, including that of Steinacker et al. (2017a) on 125 ALS patients undergoing at least a second blood sampling, show mostly stable longitudinal NFL levels (Steinacker et al., 2017a; Gille et al., 2019; Verde et al., 2019b).

### NFL in Genetic Forms of ALS

NFL in the CSF and serum of patients with genetic forms of ALS has been investigated by few studies in the past. In 2007, Zetterberg et al. (2007) reported lower CSF NFL levels in patients with both familial and apparently sporadic ALS harboring mutations in the *SOD1* gene, although NFL did not differ in general between familial and sporadic ALS cases of the cohort. While other studies did not find significant differences in CSF or serum NFL levels between patients with mutations in *SOD1*, *TARDBP*, or *FUS* or the HRE of *C9orf72* and sporadic cases (Weydt et al., 2016; Verde et al., 2019b), a recent larger study reported higher levels in patients with the *C9orf72* HRE (Benatar et al., 2020). Weydt et al. (2016) investigated CSF and serum NFL (as well as CSF pNFH) in a cohort of ALS patients carrying disease-causing mutations, related presymptomatic mutation carriers, and healthy non-carriers belonging to the same families. With limitations due to the cross-sectional nature of their study, they found that NFL (and pNFH) levels in presymptomatic mutation carriers did not differ from those in non-carriers, while elevated levels were consistently observed in symptomatic mutation carriers, starting shortly after symptom onset.

In their fundamental work, Benatar et al. (2018) conducted a longitudinal investigation of CSF and serum NFL levels in the large North-American *Pre-fALS* cohort of familial ALS cases and related presymptomatic ALS gene mutation carriers, thus overcoming the limitations of the abovementioned cross-sectional study. Baseline serum NFL levels were higher in patients compared with those in both healthy controls and presymptomatic carriers (*n* = 84; 52 with *SOD1* mutations, 27 with the *C9orf72* HRE, and 5 with mutations in *TARDBP*, *FUS*, and *VCP*), with lack of significant difference between healthy controls and presymptomatic carriers. Whereas in both patients and controls serum NFL levels were stable over time, an average increase of 2.41 pg/ml per 10-year increase in age was observed in presymptomatic carriers. In the additional group of so-called converters (*n* = 10), i.e., carriers converting from presymptomatic to symptomatic disease during the study period, elevated serum NFL levels (i.e., levels above the highest value observed in controls) were found as early as 11.6 months before symptomatic conversion and continued to increase for at least 6 months after conversion. Similar patterns were observed for CSF NFL (Benatar et al., 2018). Notably, in a subsequent expansion of their study, the same investigators observed elevated CSF and serum levels of NFL in converters as far back as 6–12 months prior to phenoconversion in *SOD1* mutation carriers, as far back as 2 years in the single converter with a *FUS* mutation, and as far back as 3.5 years in the single motor converter (i.e., developing ALS) with a *C9orf72* HRE (Benatar et al., 2019).

### NFL as Pharmacodynamic Biomarker

Given its correlation with disease progression rate and its generally stable longitudinal blood levels, NFL has gained much attention as a candidate pharmacodynamic biomarker for ALS, i.e., a biomarker able to reflect target engagement by a hypothetical experimental treatment and possibly also to quantify the beneficial effect thereof. Although in general the stability of a clinical parameter over time is not a fundamental prerequisite for its validity as a marker of effectiveness of an experimental drug as demonstrated by the usefulness of normally increasing or decreasing clinical scores as outcome measures, the longitudinal stability of NFL over the natural disease course of ALS makes it easier to attribute possible changes in its levels observed during an experimental trial to an effect of the treatment itself. Moreover, it can be envisioned that in the future, when hopefully multiple effective and personalized therapies will be available for ALS patients, longitudinal changes in the levels of NFL in single patients could confirm biological response to treatment or, on the contrary, indicate the need to modify the therapeutic regimen, as is partially the case for MS (Gafson et al., 2020).

In their elegant study, Benatar et al. (2020) analyzed the theoretical performance of serum NFL as pharmacodynamic biomarker in a cohort of 220 ALS patients. In contrast to baseline pNFH, baseline serum NFL both predicts survival and improves prediction of longitudinal ALSFRS-R slope relative to the information provided by the initial ALSFRS-R slope only: these two features qualify serum NFL as a true prognostic biomarker for ALS. The biomarker shows a longitudinal increase of 0.011 log units per month (with 95% confidence interval including 0) and correlates with age increasing by 1.3% per 1-year increase in age. Importantly, thanks to its ability to predict longitudinal ALSFRS-R slope, adding baseline serum NFL to the available information at the beginning of an ALS drug trial would enable an 8.2% reduction of the cohort size necessary to detect a significant treatment effect. According to the authors, including serum NFL (but, again, not pNFH) as a pharmacodynamic biomarker would allow a much larger reduction of the sample size: indeed, in order to detect—with 90% power and a two-tailed *t*-test with 0.05 significance level—a clinically meaningful lowering in longitudinal serum NFL concentrations (i.e., corresponding to lowering from the level of fast progressors to that of slow progressors) as a sign of treatment effect, it would be necessary to enroll 64 patients, whereas 1,054 or 470 patients would be required to detect a 20 or 30% reduction in the ALSFRS-R slope, respectively (Benatar et al., 2020). It should be pointed out, however, that this comparison does not seem to be completely balanced, as in this study fast progressors and slow progressors had ALSFRS-slopes of > 1 and < 0.5 points/month, respectively: this means that reducing the slope from the level of fast progressors to that of slow progressors would imply a > 50% reduction thereof.

A practical demonstration of the concepts theorized by Benatar et al. (2018) comes from the recent study of Dorst et al. (2020), who retrospectively analyzed serum NFL levels in ALS patients who had been enrolled in the LIPCAL-ALS trial of high-caloric fatty diet (HCFD). This trial had had an overall lack of effect on survival, but *post hoc* analysis had demonstrated a beneficial effect on patients with fast disease progression (Ludolph et al., 2020). Indeed, analysis of serum NFL levels demonstrated that while in the placebo group of the trial serum NFL levels had increased longitudinally, in HCFD-treated patients, serum NFL had decreased, and this difference was attributable to an effect in patients with fast progression, i.e., those benefiting from treatment in terms of survival. Moreover, within the subgroup of patients with baseline high serum NFL levels, survival was prolonged in HCFD-treated patients compared with those receiving placebo, and also in this subgroup, a corresponding longitudinal decrease of serum NFL levels was observed in the former compared with the latter (Dorst et al., 2020). Finally, NFL measurement was included in the historic phase 1–2 trials of the anti-SOD1 antisense oligonucleotide tofersen in patients with ALS due to *SOD1* mutations, whose results were published in 2020. The highest dose of the drug administered intrathecally over a period of 12 weeks produced a significant decrease in CSF SOD1 concentrations; importantly, this was accompanied by a decrease of both CSF and plasma concentrations of NFL (and pNFH), representing one of the first examples of the use of neurofilaments as pharmacodynamic biomarkers for an ALS treatment trial (Miller et al., 2020).

## NFL as Biomarker for Frontotemporal Dementia

### NFL as FTD Diagnostic Biomarker

The first reports of NFL elevations in the CSF of patients with FTD are those of Rosengren et al. (1999) and Sjögren et al. (2000). In particular, Rosengren et al. (1999) demonstrated higher CSF NFL levels in patients with FTD, AD, and vascular dementia (VaD) in comparison with healthy individuals and hypothesized that raised NFL levels were the consequence of degeneration of the brain white matter in those diseases (Table 3). More than 10 years later, Scherling et al. (2014) measured NFL in the CSF of 79 patients with the three forms of FTD [bvFTD, nfvPPA (there called progressive non-fluent aphasia), and svPPA (there called semantic dementia)] and found increased levels compared with both controls and patients with AD. The difference between FTD and AD remained significant also if the comparison was limited to the 44 FTD patients with increased level of confidence of FTLD pathology (due to the presence of a FTLD-causing genetic mutation, autopsy neuropathological evidence, or negative result of amyloid PET) vs. the 14 AD patients with increased level of confidence of amyloid pathology (positive amyloid PET or neuropathological evidence). Notably, a recent study on neuropathologically confirmed cases showed that higher CSF NFL levels are still observed in FTLD in comparison with AD even after excluding cases of FTLD-ALS from the analysis (Cousins et al., 2020).

**TABLE 3:** Representative studies of NFL as biomarker for FTD.

Study (authors and year)	Patients	Biological fluid	Type of assay	Main findings
Rosengren et al. (1999)	5 patients with FTD, 39 HCs	CSF	ELISA	CSF NFL levels are increased in ALS compared with HCs.
Scherling et al. (2014)	79 FTD patients (45 bvFTD, 18 nfvPPA, 16 svPPA), 8 presymptomatic carriers of FTD-causing gene mutations, 22 PSP patients, 50 AD patients, 6 PD patients, 17 CBS patients, 47 HCs	CSF	ELISA	CSF NFL levels are higher in all FTD subgroups compared with HCs, AD patients, presymptomatic carriers of FTD mutations, and PD patients. CSF NFL in all FTD subgroups correlates moderately with CDR-SB (*r* = 0.359). CSF NFL has moderate negative correlations with MMSE score (*r* = -0.549) and with the performance in several neuropsychological tests (mostly of frontal-executive functions). In FTD and bvFTD, CSF NFL levels correlate negatively with gray matter volume of frontal, temporal, parietal, occipital, and cingulate cortices and, to a lesser extent, with volume of associated white matter.
Steinacker et al. (2017b)	99 PPA patients (40 nfvPPA, 38 svPPA, 21 lvPPA), 35 HCs	Serum, CSF	ECL (serum), ELISA (CSF)	Serum NFL levels are higher in each PPA variant compared to HCs. Both nfvPPA and svPPA have higher levels compared with lvPPA. Similar findings for CSF NFL. Performance in discriminating between PPA and HCs: sensitivity 95%, specificity 70%. Performance in discriminating between nfvPPA + svPPA vs. lvPPA: sensitivity 81%, specificity 67%. Longitudinal serum NFL (subcohort of 37 PPA patients): increase in nfvPPA and svPPA, no significant change in lvPPA. In the whole PPA cohort, longitudinal increase of serum NFL correlates with longitudinal atrophy progression in left frontal lobe. In patients with nfvPPA and svPPA, longitudinal increase of serum NFL correlates with longitudinal atrophy progression in right middle frontal gyrus. Longitudinal serum NFL change in PPA correlates moderately with longitudinal change in CDR-SB and, in nfvPPA and svPPA, also with longitudinal change in CDR-FTD-SB.
Meeter et al. (2018b)	361 FTD patients (179 bvFTD, 17 FTD-MND, 36 svPPA, 19 nfvPPA, 4 lvPPA, 42 CBS, 64 PSP), 45 HCs. Definite pathology known in 68 patients (49 FTLD-TDP, 18 FTLD-tau, 1 FTLD-FUS)	CSF	ELISA	All clinical entities except for lvPPA have higher CSF NFL levels compared with HCs, with the strongest elevation in FTD-MND. CSF NFL is higher in FTD-MND compared with bvFTD. Performance in discriminating between FTD patients and controls: AUC 0.87, sensitivity 79%, specificity 89%. CSF NFL levels do not differ significantly between pathology-proven FTLD-tau and FTLD-TDP, but when clinically suspected cases of the two types are added, FTLD-TDP has higher CSF NFL levels compared with FTLD-tau. CSF NFL correlates moderately with CDR-SB and weakly with FAB and (negatively) with MMSE. FTD patients with *GRN* mutations have higher CSF NFL levels than those with *C9orf72*, *MAPT*, or no mutations. CSF NFL levels are negatively associated with survival in FTD and bvFTD.
Steinacker et al. (2018)	74 bvFTD patients, 26 AD patients, 17 MCI patients, 15 HCs	Serum, CSF	Simoa (serum), ELISA (CSF)	In bvFTD, serum and CSF NFL correlate strongly (*r* = 0.706). CSF NFL levels are higher in bvFTD compared with AD and MCI. Serum NFL is higher in bvFTD compared with AD, MCI, and HCs. Diagnostic performance of serum NFL for bvFTD: vs. AD: AUC 0.6762; vs. MCI: AUC 0.9094; vs. HCs: AUC 0.8514. In bvFTD, serum NFL correlates moderately with CDR-SB (*r* = 0.4402) and CDR-FTD-SB (*r* = 0.5297) and negatively with MMSE (*r* = -0.3242). In bvFTD, serum NFL has a moderate negative correlation with volumes of the frontal lobe (*r* = -0.5857), striatum (*r* = -0.5244), right amygdala (*r* = -0.4951), and frontal lobe white matter (*r* = -0.5382). In bvFTD, serum NFL increases at follow-up (subcohort of 64 patients).
van der Ende et al. (2019)	59 FTD patients with *MAPT* (*n* = 10), *GRN* (*n* = 25), or *C9orf72* (*n* = 24) mutations, 149 presymptomatic mutation carriers (24 *MAPT*, 79 *GRN*, 46 *C9orf72*), 127 non-carrier relatives	Serum	Simoa	Serum NFL is higher in symptomatic mutation carriers compared with presymptomatic carriers (AUC for discrimination: 0.93) and non-carriers (AUC: 0.95). Symptomatic *GRN* mutation carriers have higher serum NFL levels compared with symptomatic *MAPT* and *C9orf72* mutation carriers. Serum NFL strongly correlates with age in the whole cohort (*r* = 0.770) and in non-carriers (with an estimated increase of 1.2% per year). Serum NFL levels do not differ between presymptomatic carriers and non-carriers in general, but do from the age of 48 years onward. Longitudinal serum NFL levels are stable in non-carriers but increase in presymptomatic carriers (which is due to *C9orf72* mutation carriers). The rate of serum NFL increase is higher in converters than in non-converters. Among presymptomatic carriers, baseline serum NFL discriminates between converters (*n* = 9; 6 *GRN*, 2 *MAPT*, 1 *C9orf72*) and non-converters (AUC: 0.93). Among symptomatic carriers, longitudinal serum NFL levels are stable in *MAPT* and *C9orf72* mutation carriers but increase in *GRN* mutation carriers. Across all groups, the rate of serum NFL change is associated with the change in volume of the frontal lobe, insula, cingulate gyrus, hippocampus, putamen, whole brain, temporal lobe, amygdala, and cerebellum and with change in MMSE over time.
Katisko et al. (2020)	91 FTD patients (66 bvFTD, 16 nfvPPA, 4 svPPA, 5 FTD-MND), 34 patients with primary psychiatric disorders (psychoses and/or mood disorders)	Serum	Simoa	Serum NFL discriminates between FTD and primary psychiatric disorders with AUC 0.850 (sensitivity 80%, specificity 85%) and between bvFTD and primary psychiatric disorders with AUC 0.830 (sensitivity 79%, specificity 85%).
Benussi et al. (2020)	291 patients with FTLD syndromes (134 bvFTD, 48 nfvPPA, 27 svPPA, 51 CBS, 31 PSP), 63 AD, 63 HCs	Serum	Simoa	Serum NFL levels are higher in bvFTD, nfvPPA, and svPPA compared with HCs. Serum NFL discriminates between FTLD syndromes and HCs with AUC 0.862 (sensitivity 71.5%, specificity 92.1%). Serum NFL levels are higher in nfvPPA compared with svPPA. Serum NFL levels in FTLD syndromes are higher in patients with *GRN* or *MAPT* pathogenic mutations (*n* = 30 and *n* = 3, respectively) than in those without mutations. Serum NFL levels weakly correlate with several measures of functional impairment, cognitive function, and behavioral disturbance. Serum NFL levels correlate negatively with thickness of left dorsolateral prefrontal cortex. Serum NFL levels correlate with SICI and LICI (reflecting postsynaptic inhibition at the level of cortical interneurons). Higher serum NFL levels are associated with shorter survival.
Illán-Gala et al. (2021)	167 patients with FTLD syndromes (43 bvFTD, 28 nfvPPA, 18 svPPA, 36 PSP, 32 CBS, 10 ALS-FTD), of whom 70 pathology-proven (50 FTLD-tau, 18 FTLD-TDP, 2 FTLD-FUS), 43 AD patients, 55 HCs	Plasma, CSF	Simoa (plasma), ELISA (CSF)	Plasma and CSF NFL correlate strongly in FTLD syndromes (*r* = 0.82). All FTLD syndromes have higher plasma NFL levels compared with AD, with the highest levels observed in ALS-FTD. Plasma NFL discriminates very well between FTLD and HCs (AUC 0.97) but less well between FTLD and AD (AUC 0.75). Plasma NFL levels are higher in FTLD-TDP compared with FTLD-tau. In FTLD syndromes, plasma NFL has strong negative correlations with cortical thickness in frontal regions. Baseline plasma NFL is associated with faster annual worsening of CDR-FTD-SB. Plasma NFL increases over time in FTLD. Higher plasma NFL is associated with shorter survival.
Cousins et al. (2020)	27 FTLD patients, 67 AD patients (both autopsy-confirmed)	CSF	ELISA	CSF NFL is higher in FTLD compared with AD. Replacing CSF total tau with CSF NFL in the neurochemical AT(N) framework increases the accuracy of the scheme at diagnosing FTLD as suspected non-Alzheimer pathophysiology (SNAP), with sensitivity increasing from 44 to 93% and specificity remaining high at 94%.

*Relevant data with reference to NFL in FTD are reported. AD, Alzheimer’s disease; ALS, amyotrophic lateral sclerosis; bvFTD, behavioral variant of frontotemporal dementia; AUC, area under the ROC (receiver operating characteristic) curve; CBS, corticobasal syndrome; CDR-FTD-SB, Clinical Dementia Rating Scale for Frontotemporal Dementia—Sum of Boxes; CDR-SB, Clinical Dementia Rating Scale—Sum of Boxes; FAB, Frontal Assessment Battery; FTD, frontotemporal dementia; FTD-MND, frontotemporal dementia—motor neuron disease; FTLD, frontotemporal lobar degeneration; FTLD-FUS, frontotemporal lobar degeneration with FUS pathology; FTLD-tau, frontotemporal lobar degeneration with tau pathology; FTLD-TDP, frontotemporal lobar degeneration with TDP-43 pathology; HCs, healthy controls; lvPPA, logopenic variant of primary progressive aphasia; LICI, long-interval intracortical inhibition; MCI, mild cognitive impairment; nfvPPA, non-fluent variant of primary progressive aphasia; PD, Parkinson’s disease; PPA, primary progressive aphasia; PSP, progressive supranuclear palsy; SICI, short-interval intracortical inhibition; svPPA, semantic variant of primary progressive aphasia.*

In their large cohort of FTD patients [*n* = 361, actually including also patients with corticobasal syndrome (CBS) and progressive supranuclear palsy (PSP), which belong to the spectrum of FTLD syndromes], Meeter et al. (2018b) demonstrated that each clinical form of FTD had higher CSF NFL levels compared with neurologically healthy controls, with the highest levels found in FTD-MND. This resulted in an AUC of 0.87 for discriminating between FTD patients and controls (corresponding to a sensitivity of 79% and a specificity of 89% at a cutoff of 1,613 pg/ml). The only exception was represented by lvPPA, a finding which is not surprising considering that in most cases this phenotype is due to underlying AD pathology. Accordingly, both nfvPPA and svPPA show higher CSF levels of NFL compared with lvPPA, enabling discrimination between the former two entities and lvPPA with an AUC of 0.8744 in the study of Steinacker et al. (2017b). In a very large multicenter cohort of svPPA patients (*n* = 162), CSF NFL had an excellent diagnostic performance in discriminating between patients and neurologically healthy controls (AUC 0.98, sensitivity 93%, specificity 98%) (Meeter et al., 2019).

CSF NFL is higher also in FTD cases compared with cases of AD with early onset (≤ 65 years), with one study reporting an AUC of 0.80 for the discrimination between the two, which is clinically meaningful considering the higher prevalence of FTD in presenile dementia (de Jong et al., 2007). In a cohort in which the diagnostic certainty was increased based on genetic, neuropathological, or CSF AD biomarker data or, in the case of FTLD, on the co-occurrence of ALS, given the differences in CSF NFL levels between patients with FTLD (showing the highest levels), patients with AD (intermediate levels), and cognitively normal controls (lowest levels), CSF NFL was able to correctly assign 85.2% of patients to each of the three categories (Alcolea et al., 2017). CSF NFL is higher in FTD compared also with dementia with Lewy bodies (DLB), VaD, mixed dementia (i.e., dementia with both Alzheimer and vascular pathology), Parkinson’s disease dementia (PDD), and other forms (Skillbäck et al., 2014). The difference from DLB was also demonstrated in a cohort of pathologically confirmed cases (Olsson et al., 2019). Conversely, CSF NFL is lower in FTD than in CJD (Antonell et al., 2020). Olsson et al. (2019) showed that adding CSF NFL to a neurochemical diagnostic algorithm based on the three classical CSF biomarkers Aβ_1__–__42_, total tau, and phosphorylated tau increased diagnostic accuracy for the discrimination between FTD and healthy controls from 63 to 81%. The increased CSF levels of NFL in FTD have been confirmed by meta-analyses examining studies conducted on > 1,800 FTD patients, with ratios of means of 3.41 between FTD and cognitively healthy controls, 2.08 between FTD and AD, 2.50 between FTD and DLB, and 1.56 between FTD and VaD (Bridel et al., 2019; Forgrave et al., 2019). In their recent work on a cohort of neuropathologically confirmed FTLD and AD cases (*n* = 27 and *n* = 67, respectively), Cousins et al. (2020) examined the result of including CSF NFL instead of CSF total tau in the neurochemical AD AT(N) framework (based on CSF levels of Aβ_1__–__42_, total tau, and phosphorylated tau) in order to correctly classify FTLD cases as SNAP (suspected non-Alzheimer pathophysiology). Importantly, the replacement of total tau with NFL improved the diagnostic classification, with sensitivity rising from 44 to 93% and specificity remaining high at 94%. This elegant work might pave the way for the introduction of CSF NFL in the AT(N) framework in the near future, thus increasing correct pathological attribution of cases of cognitive impairment, with relevant consequences for diagnosis and inclusion in clinical trials.

In the last 5 years, NFL has been increasingly studied in peripheral blood in FTD. Given the strong correlation between serum and CSF levels of NFL, Wilke et al. (2016) demonstrated that serum NFL was higher in FTD patients compared with that in neurologically healthy controls and that the diagnostic performance of the blood biomarker for the discrimination between the two conditions was similar to that of CSF NFL (AUCs, 0.81 for serum and 0.88 for CSF, respectively). Indeed, the recent study of Benussi et al. (2020) on a large cohort of FTD patients (*n* = 291, actually including also patients with CBS and PSP) reported an AUC as high as 0.862 for serum NFL in distinguishing FTD patients from healthy controls. The sensitivity and specificity for discriminating between FTD and healthy controls are as high as 84 and 96%, respectively, and serum NFL is higher in all three clinical forms of FTD, i.e., bvFTD, svPPA, and nfvPPA, compared with controls (Rohrer et al., 2016). CSF and serum NFL levels strongly correlate with each other also when limiting the analysis to bvFTD cases (Steinacker et al., 2018). According to the work of Steinacker et al. (2018) on 74 bvFTD cases, serum NFL enables the discrimination of this condition from neurologically healthy controls, patients with MCI, and patients with AD with sensitivities of 91, 74, and 74%, respectively, and specificities of 79, 74, and 58%, respectively. The same group measured serum NFL in 99 patients with primary progressive aphasia (PPA) and showed it to be higher in each of the three categories of PPA (nfvPPA, svPPA, and lvPPA), enabling discrimination between patients with PPA and neurologically healthy controls with 95% sensitivity and 70% specificity (Steinacker et al., 2017b). Similar to what was observed for CSF NFL, serum NFL was able to distinguish nfvPPA and svPPA from lvPPA (Steinacker et al., 2017b), while, in another cohort, nfvPPA was reported to have higher serum NFL levels than svPPA (Benussi et al., 2020). The already mentioned meta-analysis of Forgrave et al. (2019) conducted in 2019 reported a ratio of means of serum NFL between FTD patients and cognitively normal controls of 2.65.

In their recently published study, Illán-Gala et al. (2021) measured plasma NFL in a large cohort of FTD patients (*n* = 167, including CBS and PSP), of whom 70 had neuropathological confirmation. While not correlating with plasma tau, plasma NFL strongly correlated with CSF NFL. Plasma NFL was higher in clinically diagnosed FTD syndromes than in clinically diagnosed AD and in neurologically healthy controls and had an excellent performance in discriminating between FTD syndromes and controls (AUC 0.97), but was less good in discriminating between FTD syndromes and clinically diagnosed AD (AUC 0.75). Notably, in the subcohort of neuropathologically confirmed FTD cases, plasma NFL was higher both in FTLD-TDP and in FTLD-tau compared with AD cases with neuropathological or PET evidence of amyloid pathology.

A special issue regarding NFL as diagnostic biomarker for FTD is represented by the differentiation from primary psychiatric disorders (PPDs), which can present with similar behavioral features and be accompanied by cognitive impairment. Importantly, patients with bvFTD were shown to have higher CSF NFL levels compared with those suffering from PPDs, enabling discrimination between the two categories with AUC as high as 0.93 (Vijverberg et al., 2017). Similar results were obtained by Katisko et al. (2020) for serum NFL measured in a cohort of 91 FTD patients and 34 patients with PPDs. A study investigating the diagnostic performance of serum NFL in discriminating between bvFTD and specific psychiatric diseases reported AUCs of 0.89 for depression, 0.94 for bipolar disorder, and 0.90 for schizophrenia (Al Shweiki et al., 2019). Given these promising findings, a consensus paper of the recently established Neuropsychiatric International Consortium for Frontotemporal Dementia (NIC-FTD) highlights the potential role of CSF and serum NFL for distinguishing bvFTD from psychiatric disorders (Ducharme et al., 2020).

### Relationship With Underlying Neuropathology and Presence of ALS

Higher CSF NFL levels have been reported in cases of probable or definite FTLD with TDP-43 pathology (FTLD-TDP) compared with cases of FTLD with tau pathology (FTLD-tau), whereby definite cases are pathologically proven or carry a pathology-causing mutation (*GRN* or *C9orf72* for FTLD-TDP, *MAPT* for FTLD-tau) while probable cases are clinically defined (with FTD-MND and svPPA pointing to TDP-43 pathology and PSP and CBS pointing to tau pathology). CSF NFL may have an AUC as high as 0.861 for the discrimination between definite and probable cases of the two pathological categories, corresponding to 80% sensitivity and 81% specificity (Abu-Rumeileh et al., 2018). Probable or definite FTLD-TDP has been reported to have higher CSF NFL levels than FTLD-tau not only when accompanied by ALS but also in the absence of the motor phenotype (Pijnenburg et al., 2015; Abu-Rumeileh et al., 2018). Other studies, however, did not confirm in genetically or neuropathologically defined cases the difference which is observed when including phenotypically defined ones (Goossens et al., 2018; Meeter et al., 2018b). On the contrary, the recent investigation of Illán-Gala et al. (2021) demonstrated higher plasma NFL levels in pathology-proven FTLD-TDP cases compared with pathological FTLD-tau cases, a difference which held true also after excluding cases with ALS-FTD from the FTLD-TDP neuropathological cohort. In agreement with the tendency of FTLD-TDP toward higher NFL levels is the demonstration, in the large neuropathological investigation of Olsson et al. (2019), of a correlation between CSF NFL and TDP-43 load in 13 of 17 brain regions in the whole neuropathological cohort (*n* = 120) encompassing several neurodegenerative diseases.

Patients with ALS have higher median NFL levels both in CSF and in serum compared with those with FTD (Wilke et al., 2016; Gaiani et al., 2017; Skillbäck et al., 2017; Verde et al., 2019b). ALS-FTD is the form of FTD with the highest NFL levels both in CSF and in plasma (Meeter et al., 2018b; Illán-Gala et al., 2021). Accordingly, in the large FTD cohort of Meeter et al. (2018b) (total FTD cohort, *n* = 361; ALS-FTD subcohort, *n* = 17), ALS-FTD had significantly higher CSF NFL levels compared with bvFTD. An investigation of the Sant Pau Initiative on Neurodegeneration (SPIN) shows that the biomarker could enable diagnosis of ALS within a cohort of FTD patients with AUC of 0.705 (Delaby et al., 2020). The same study showed a gradient of CSF NFL concentrations with the highest levels in ALS, intermediate levels in ALS-FTD, and lowest levels in FTD; however, the only statistically significant differences were those between ALS and FTD and between ALS and ALS-FTD, whereas ALS-FTD did not significantly differ from FTD alone (Delaby et al., 2020). However, not all studies have found a significant difference in CSF NFL levels between ALS and ALS-FTD (Steinacker et al., 2016; Illán-Gala et al., 2018).

In studies differentiating FTD cases according to definite (pathologically proven or carrying a gene mutation) or probable (inferred from phenotype) pathological subtype, there is no agreement as to whether FTLD-TDP cases with ALS have higher CSF NFL levels than FTLD-TDP cases without ALS (Pijnenburg et al., 2015; Abu-Rumeileh et al., 2018). In the investigation of Olsson et al. (2019), although in the clinical cohort ALS patients had higher CSF NFL levels than FTD patients, among pathologically confirmed cases, ALS showed only a trend toward higher levels compared with FTD, without reaching statistical significance (Olsson et al., 2019). Notably, among patients with FTD with the *C9orf72* HRE, those with ALS-FTD were shown to have higher CSF NFL levels compared with those with FTD alone (Meeter et al., 2018a).

### Relationship With Clinical Features and Longitudinal Kinetics

In a cohort of autopsy-confirmed FTLD cases, no relationship was found between sex and CSF NFL levels (Cousins et al., 2020). However, a very recent study on a large cohort comprising patients with FTD and asymptomatic individuals with a family history of FTD (*n* = 277) found higher plasma NFL levels in women even after correction for disease severity, age, and clinical phenotype (Rojas et al., 2021). While CSF NFL levels correlate with age in neurologically healthy controls and in patients with AD, no such correlation is observed in FTD patients (Skillbäck et al., 2014; Goossens et al., 2018), a finding confirmed by the meta-analysis of Bridel et al. (2019). The same lack of correlation with age in FTD was found in the recent investigation of Illán-Gala et al. (2021) on plasma NFL. Pertaining to FTD clinical subtypes, no correlation between serum NFL and age was found in bvFTD by Steinacker et al. (2018), whereas the same group observed a weak correlation between serum NFL and age at onset in all forms of PPA together as well as in nfvPPA and svPPA considered individually, while the correlation was stronger for lvPPA, which, again, is not surprising considering that AD pathology most commonly underlies this clinical variant (Steinacker et al., 2017b). Although Meeter et al. (2016, 2018b) reported a weak association of CSF NFL with disease duration at sampling in their large FTD cohort, the finding has not been replicated in studies investigating serum NFL in bvFTD and PPAs and CSF or serum NFL in genetic forms of FTD (Meeter et al., 2016, 2018b; Steinacker et al., 2017b, 2018; van der Ende et al., 2019).

NFL concentrations in the CSF and blood in FTD patients are associated with cognitive features. Although not according to all studies, CSF NFL concentrations negatively correlate with the score in the Mini Mental State Examination (MMSE) (Sjögren et al., 2000; Scherling et al., 2014; Skillbäck et al., 2014); in the study of Scherling et al. (2014), this is true also when considering only the cases with increased level of certainty of FTLD pathology (due to autopsy evidence, presence of gene mutations, or negative result of amyloid PET scan). CSF NFL also correlates with the score of the Clinical Dementia Rating Scale—Sum of Boxes (CDR-SB) in FTD as a whole and in the three subclasses bvFTD, nfvPPA, and svPPA, as well as, in bvFTD, with the score of the modified version of the CDR for FTD (CDR-FTD-SB) (Scherling et al., 2014; Ljubenkov et al., 2018). CSF NFL is weakly associated with the score of the Frontal Assessment Battery (FAB) in FTD and correlates with the scores of several neuropsychological tests, especially regarding frontal-executive functions, both in FTD and in the subclasses bvFTD and nfvPPA (Scherling et al., 2014; Meeter et al., 2018b). In svPPA, the biomarker shows a weak negative correlation with the score of the Boston Naming Test (Meeter et al., 2019). CSF NFL has also relationships with longitudinal cognitive data: CSF NFL at baseline correlates with worsening of the MMSE score at follow-up evaluation in FTD and with worsening of CDR-FTD-SB score and other neuropsychological parameters both in bvFTD and in nfvPPA (Ljubenkov et al., 2018; Olsson et al., 2019). For blood NFL, similar associations were reported. In the large cohort of Benussi et al. (2020), serum NFL correlated with functional impairment, dementia severity as measured with the CDR-FTD-SB, performance in several cognitive tests (especially regarding frontal-executive functions), and behavioral alterations. Also, in the subclass bvFTD serum, NFL correlates with the CDR-FTD-SB, as well as with the traditional CDR-SB (Steinacker et al., 2018). In svPPA, plasma NFL correlates with neuropsychological measures of semantic impairment (Heller et al., 2020a). Regarding longitudinal associations, plasma NFL at baseline correlates with worsening of CDR-FTD-SB score both in FTD as a whole and in the subclasses bvFTD, svPPA, and ALS-FTD (Illán-Gala et al., 2021).

Most investigations agree that CSF NFL is associated with survival, with Meeter et al. (2018b) reporting a HR for tertiles of CSF NFL concentrations of 1.7. The association is true also when limiting the analysis to bvFTD patients or to FTD patients with definite or probable FTLD-TDP pathology based on neuropathology, genetics, or phenotype (Pijnenburg et al., 2015; Skillbäck et al., 2017). Importantly, NFL is associated with survival in FTD also when measured in serum or in plasma (Benussi et al., 2020; Illán-Gala et al., 2021).

Although most studies on longitudinal NFL levels in FTD have been conducted on blood because of the less invasive nature of blood sampling compared with CSF sampling, two investigations reported longitudinal data regarding CSF NFL: whereas in the 27 patients with a follow-up lumbar puncture in the cohort of Ljubenkov et al. (2018) no consistent longitudinal trend of NFL could be recognized, 11 of the 14 FTD patients with a longitudinal CSF sample studied by Skillbäck et al. (2017) showed higher levels of NFL compared with the first sample (Skillbäck et al., 2017; Ljubenkov et al., 2018). Longitudinal studies on blood NFL enable investigation of larger cohorts. The group of Steinacker et al. (2017b, 2018) reported an increase in serum NFL at follow-up 1 year after baseline sampling in bvFTD as well as in nfvPPA and svPPA, while such an increase was not observed in lvPPA. In nfvPPA and svPPA, longitudinal change in serum NFL correlates with longitudinal worsening of the CDR-FTD-SB score (Steinacker et al., 2017b). An increase at follow-up was also observed for plasma NFL in FTD in the recent study of Illán-Gala et al. (2021).

### NFL in Genetic Forms of FTD

NFL has been investigated in patients with genetic FTD, i.e., those carrying mutations in the three main genes *MAPT*, *GRN*, and *C9orf72*. Most studies on CSF NFL report higher levels in patients with *GRN* mutations compared with patients without known gene mutations, patients with *MAPT* mutations or the *C9orf72* HRE, or patients with FTLD-tau as neuropathologically or genetically defined (Meeter et al., 2016, 2018b; Goossens et al., 2018).

Results of studies on blood NFL are less clear-cut, with some investigations not reporting significant differences in serum NFL between bvFTD cases with and without gene mutations or between FTD patients with mutations in different genes (Rohrer et al., 2016; Steinacker et al., 2018). Moreover, higher serum or plasma NFL levels have been reported in FTD patients with the *C9orf72* HRE compared with FTD patients without mutations, who, in turn, do not differ from patients with *GRN* or *MAPT* mutations (Cajanus et al., 2020; Illán-Gala et al., 2021). Higher plasma NFL levels have also been reported in FTD patients with the *C9orf72* HRE compared with patients with *MAPT* mutations (Heller et al., 2020b). Notwithstanding this, also regarding blood NFL, several investigations reported higher serum or plasma levels in patients with *GRN* mutations as compared with patients without mutations in known genes, patients with *MAPT* mutations, or patients with the *C9orf72* HRE (including patients with ALS-FTD) (Meeter et al., 2016; van der Ende et al., 2019; Benussi et al., 2020; Heller et al., 2020b; Rojas et al., 2021).

The relationship between NFL and cognitive impairment has also been investigated in cohorts of genetic FTD. In patients with mutations in the three main genes *MAPT*, *GRN*, and *C9orf72*, both CSF and serum NFL correlated with the score of the CDR-SB (Meeter et al., 2016). In a cohort of patients with the *C9orf72* HRE, CSF NFL correlated both with CDR-SB score and, negatively, with the MMSE score (Meeter et al., 2018a). Also in cohorts of FTD patients carrying mutations in the three genes, both CSF and serum NFL were associated with survival (Meeter et al., 2016, 2018a; Cajanus et al., 2020). Pertaining to longitudinal kinetics, in FTD patients carrying mutations in the three genes as a whole, serum NFL does not show a consistent change over time, but when analyzing patients with the three genetic forms separately, an increase is observed in *GRN* mutation carriers but not in the other two groups (van der Ende et al., 2019).

Several important studies conducted by centers collaborating in the Genetic FTD Initiative (GENFI) have investigated NFL in presymptomatic carriers of FTD-causing gene mutations. Meeter et al. (2016) measured NFL in the CSF of 86 FTD patients with mutations in *MAPT* and *GRN* and the *C9orf72* HRE, 40 presymptomatic carriers of the same mutations, and 48 neurologically healthy controls. Median NFL levels were more than eight times higher in patients than in presymptomatic carriers and controls but did not significantly differ between presymptomatic carriers and controls nor between presymptomatic carriers with mutations in the three different genes. Diagnostic performance of CSF NFL in discriminating between patients and presymptomatic carriers was excellent, with AUC 0.97 and sensitivity and specificity of 84 and 100%, respectively. On the contrary, performance for the discrimination between presymptomatic carriers and controls was poor (AUC 0.65). Notably, CSF NFL correlated with age in presymptomatic carriers, and two presymptomatic *GRN* mutation carriers converting to symptomatic FTD during the study period showed a three to fourfold increase in CSF NFL levels during follow-up. Results obtained in the smaller and partially overlapping cohort in which NFL was measured in serum were similar, in agreement with the strong correlation observed between CSF and serum NFL levels (*r* = 0.87) (Meeter et al., 2016). Similar results for CSF NFL were obtained for carriers of the *C9orf72* HRE (Meeter et al., 2018a). Two investigations on the Danish FTD-3 family with the rare genetic form of FTD due to mutation of the gene *CHMP2B* demonstrated higher levels of CSF and serum NFL not only in symptomatic carriers of the mutation (*n* = 12 in the larger and more recent study) compared with both presymptomatic carriers (*n* = 10) and healthy non-carriers (*n* = 16), but also in presymptomatic carriers compared with non-carriers (Rostgaard et al., 2018; Toft et al., 2021).

In their fundamental study, van der Ende et al. (2019) from the GENFI analyzed serum NFL in a large cohort (*n* = 140) of presymptomatic carriers of *MAPT* and *GRN* mutations and the *C9orf72* HRE, 59 symptomatic carriers with FTD, and 127 neurologically healthy non-carriers belonging to the same families. Serum NFL at baseline was higher in symptomatic carriers compared with both non-carriers and presymptomatic carriers, a finding which was confirmed also when separating participants according to mutated genes. Serum NFL had a good diagnostic performance in distinguishing symptomatic from asymptomatic carriers, with AUC 0.93, corresponding to 86% sensitivity and 87% specificity. Although in general serum NFL at baseline did not significantly differ between presymptomatic carriers and non-carriers, when including age in the analysis, a significant difference was observed, with presymptomatic carriers showing higher serum NFL levels after the age of 48 years. Importantly, serum NFL at baseline was higher in the nine presymptomatic carriers who converted to symptomatic disease during the follow-up period of the study in comparison with non-converters, enabling good discrimination at baseline between converters and non-converters, with AUC 0.93 and corresponding sensitivity and specificity of 100 and 84%, respectively. As expected, in the follow-up period, an increase in serum NFL was observed in converters. An increase in serum NFL was observed also in the whole group of non-converting presymptomatic carriers, but separate analyses conducted according to gene mutations showed that this increase was attributable to cases carrying the *C9orf72* HRE and not to cases with *MAPT* or *GRN* mutations. Not surprisingly, the rate of serum NFL increase during follow-up was higher in converters compared with non-converting presymptomatic carriers (van der Ende et al., 2019). A very recent study of the GENFI including presymptomatic and symptomatic *GRN* mutation carriers investigating the temporal cascade of multimodal biomarkers by means of discriminative event-based modeling (DEBM) demonstrated that, both in bvFTD and in nfvPPA, serum NFL is—together with language—the first biomarker to become abnormal in this genetic form of FTD (Panman et al., 2021).

Heller et al. (2020b) investigated plasma NFL in a large cohort comprising 196 presymptomatic and 90 symptomatic carriers of *GRN* and *MAPT* mutations and the *C9orf72* HRE as well as 183 neurologically healthy non-carriers belonging to the same families. As expected, for each of the three genes, symptomatic carriers had higher plasma NFL levels than presymptomatic carriers. Additionally, plasma NFL was higher in presymptomatic carriers of the *C9orf72* HRE compared with non-carriers, whereas no statistically significant difference was observed between presymptomatic carriers of *MAPT* or *GRN* mutations and non-carriers (Heller et al., 2020b). A very recent longitudinal study on plasma NFL examined both a cohort from the GENFI (*n* = 297) and one from the similar North-American network LEFFTDS/ARTFL (*n* = 277), including patients with mild cognitive impairment (MCI) or mild behavioral impairment (MBI) as defined by a score of 0.5 in the CDR-FTD: in the original cohort (from the LEFFTDS/ARTFL), baseline plasma NFL was higher in asymptomatic patients converting to MCI/MBI or to dementia in the next 2 years than in non-converters, and in the validation cohort (from the GENFI), asymptomatic participants or mildly symptomatic ones (MCI/MBI) had higher plasma NFL at baseline compared with corresponding non-converting individuals. However, baseline plasma NFL discriminated only poorly between asymptomatic and MCI/MBI participants (AUCs: 0.676 in the original cohort and 0.641 in the validation cohort) (Rojas et al., 2021).

### Relationship With Neuroimaging

Several studies have investigated the relationship between CSF or blood NFL and magnetic resonance imaging (MRI) data, in most cases finding correlations between NFL and brain atrophy, particularly in frontal and temporal lobes. In the cohort of Scherling et al. (2014), CSF NFL in FTD and in bvFTD correlated negatively with gray matter volume of frontal, temporal, parietal, occipital, and cingulate cortices and, to a lesser extent, with the volume of the white matter associated with most of these regions. In FTD, serum NFL has been reported to correlate inversely with thickness of the prefrontal, temporal, and parietal cortices (Benussi et al., 2020). In their work on bvFTD, Steinacker et al. (2018) demonstrated negative correlations between serum NFL and volumes of frontal lobe, striatum, right amygdala, and frontal lobe white matter. The negative correlation with cortical thickness of frontal regions in FTD was recently confirmed also for plasma NFL (Illán-Gala et al., 2021).

Other studies investigated the relationship between baseline NFL levels and longitudinal changes in brain MRI in FTD or bvFTD, finding an association of CSF NFL with faster frontotemporal volume loss and faster decline in frontotemporal FA in bvFTD and correlations of serum NFL with atrophy rates of the frontal lobe as well as of the thalamus, caudate, putamen, pallidus, and overall subcortical white matter (Rohrer et al., 2016; Ljubenkov et al., 2018; Cajanus et al., 2020). Interestingly, in their large FTD cohort, Benussi et al. (2020) also reported a correlation of serum NFL with the neurophysiological measures, obtained with transcranial magnetic stimulation (TMS), short-interval intracortical inhibition (SICI), and long-interval intracortical inhibition (LICI), reflecting short-lasting postsynaptic inhibition mediated by GABA A and B receptors, respectively, at the level of cortical interneurons.

In svPPA, CSF NFL was reported to negatively correlate with gray matter volume of the parahippocampal gyrus of the dominant atrophic side, while for plasma NFL, a negative correlation was observed with gray matter volume of the combined temporal lobes (Meeter et al., 2019; Heller et al., 2020a). In nfvPPA, baseline CSF NFL was shown to be associated with faster frontotemporal volume loss and faster decline in frontotemporal FA (Ljubenkov et al., 2018). Pertaining to longitudinal NFL data, Steinacker et al. (2017b) reported correlations of increases in serum NFL at follow-up with atrophy rate of the left frontal lobe in patients with PPA and with atrophy rate of the right middle frontal gyrus in the subcohort of patients with nfvPPA and svPPA.

In the study of Meeter et al. (2016) from the GENFI on patients with FTD carrying mutations in the three main genes *MAPT*, *GRN*, and *C9orf72*, NFL correlated negatively with volumes of the whole brain, frontal cortex, and insular cortex. In presymptomatic carriers, NFL correlated with volumes of the whole brain and frontal, temporal, and parietal cortices. In mutation carriers with follow-up MRI data, baseline CSF NFL correlated with atrophy rate of whole brain and of frontal, temporal, parietal, insular, and cingulate cortices. In the large investigation of van der Ende et al. (2019) on serum NFL in carriers of FTD gene mutations, negative correlations were observed at baseline between NFL and mostly frontotemporal brain volumes; moreover, in the whole cohort, the rate of change of serum NFL over time was associated with the longitudinal change in the volume of the whole brain and in the volumes of the frontal lobe, insula, cingulate gyrus, hippocampus, putamen, temporal lobe, amygdala, and cerebellum.

## Conclusion and Perspectives

A large body of evidence demonstrates that NFL is increased in the CSF and, consequently, in the blood, in both ALS and FTD in comparison with normal conditions (Scherling et al., 2014; Steinacker et al., 2016; Gille et al., 2019; Illán-Gala et al., 2021). In the case of ALS, the diagnostic potential of CSF and blood NFL is not much lower than that of the established CSF biomarkers for Alzheimer’s disease (Blennow et al., 2010; Steinacker et al., 2016; Verde et al., 2019b). Therefore, it seems reasonable to consider the introduction of NFL in diagnostic criteria for ALS as a supportive element, with measurement in blood substituting for that in CSF when a lumbar puncture cannot be performed. Blood NFL measurement could also be implemented in the future as a large-scale screening test on individuals complaining of early neuromuscular symptoms (e.g., fasciculations), in order to identify those with “abnormal” values deserving prioritization for specialized evaluation (Verde et al., 2019b). However, the putative added value of NFL for the diagnosis of ALS should be evaluated by means of prospective studies including assessment of benefits for patients and the healthcare system. Measurement of NFL could be most beneficial in individuals at genetic risk for ALS—and for FTD—enabling optimization of the timing of prophylactic/therapeutic interventions aimed at opposing the disease process still in its subclinical phase (Benatar et al., 2018; van der Ende et al., 2019). At least as promising as its diagnostic performance is the potential of NFL as prognostic biomarker in ALS, resulting from its correlation with the disease progression rate (Verde et al., 2019b; Abu-Rumeileh et al., 2020), its longitudinal stability (Steinacker et al., 2017a; Verde et al., 2019b), and its measurability on peripheral blood. For these reasons, NFL deserves inclusion in future treatment trials of ALS and possibly inclusion into multiparameter prognostic models.

Regarding FTD, NFL shows promising diagnostic potential for the differentiation from primary psychiatric disorders (Katisko et al., 2020); on the contrary, NFL alone does not provide enough accuracy for the differential diagnosis between FTD and other dementias on an individual patient basis (Illán-Gala et al., 2021). However, it would be informative to evaluate—possibly in prospective studies—the added value of multiparameter models including CSF or blood NFL for early diagnosis of FTD, in a similar manner to what has been shown for the incorporation of CSF NFL into the neurochemical AT(N) scheme in order to more correctly identify FTLD as SNAP (Cousins et al., 2020). The same applies for the *in vivo* differentiation between the two main neuropathologic forms of FTD (i.e., FTLD-tau and FTLD-TDP), because this would impact stratification of patients in proteinopathy-oriented drug trials and, in the future, choice of the appropriate disease-specific treatment. Moreover, as its levels correlate with several measures of disease severity in FTD, NFL deserves consideration for inclusion as pharmacodynamic biomarker in therapeutic trials for FTD, which are in general at an earlier phase of their history compared with those for ALS (Scherling et al., 2014; Boxer et al., 2020; Illán-Gala et al., 2021).

Finally, several further issues regarding NFL biology and kinetics and its role as biomarker remain incompletely solved and warrant further investigation. Examples thereof include the following:

1) It is reasonable to assume that the cause of the elevation of NFL levels in the CSF and, hence, in the blood is leakage through a damaged axonal membrane. However, it cannot be excluded that other mechanisms of emission are involved, including active secretion or exosomes (Gafson et al., 2020). Moreover, damage to the axonal membrane *per se* could not most properly explain the release of neurofilaments: rather, more proximal mechanisms could contribute, e.g., imbalances in neurofilament transport or turnover or loss of integrity of the axonal cytoskeletal scaffold. The assumption itself that neurofilaments are markers of axonal pathology could also not be totally correct, as increasing evidence points to a role of neurofilaments in synapses (Yuan et al., 2015). Finally, elevation of neurofilament levels in neurodegenerative conditions could reflect not only cell damage but also more complex pathophysiological events, as suggested by the putative etiologic role of neurofilament gene mutations in rare cases of ALS as well as by the biological impact of experimental manipulation of neurofilament genes in ALS animal models (Williamson et al., 1998).
2) Although it is generally stated that neurofilaments are released from neurons into the CNS ISF and from there pass to the CSF and hence to the blood, the actual route followed by neurofilament molecules is not completely known. A relevant role could be played by ISF drainage along intramural perivascular (mostly periarterial but also perivenous) spaces and/or by lymphatic and glymphatic routes (Albargothy et al., 2018). The relative contribution of these mechanisms could also change in different CNS diseases (Gafson et al., 2020).
3) Even the kinetics of neurofilaments in healthy conditions in the human body are not completely known. A deeper knowledge of neurofilament metabolism and turnover, e.g., by means of SILK (stable isotope labeling kinetics) studies, would be essential for complete elucidation of the potential of NFL as biomarker (Sato et al., 2018; Gafson et al., 2020).
4) It is possible that neurofilaments undergo different biochemical modifications in different pathological conditions and in different disease stages (Gafson et al., 2020). Such modifications cannot be detected by current quantitative measurement techniques but deserve investigation both for mechanistic understanding and for exploration of diagnostic–prognostic potential.
5) The controversial issue of the relationship between NFL elevation and the extent of UMN vs. LMN degeneration in ALS should be clarified. This will require investigations using homogeneous methods, longitudinal observations, and large cohorts. An aid could also be offered by the development of assays specific for neurofilament forms reflecting CNS vs. PNS pathology, e.g., targeting α-internexin or peripherin, but also of hypothetical assays capable of recognizing biochemical differences which could exist between NFL forms released by UMNs and LMNs (Sato et al., 2018; Gafson et al., 2020).
6) The notion that NFL measurement is useful in the differential diagnosis of ALS is derived from studies on cohorts of ALS mimics which are admittedly quite large but are heterogeneous, including several forms of UMN, LMN, or related diseases (Verde et al., 2019b). To fully elucidate the usefulness of the biomarker in this context, it would be highly informative to compare ALS patients with large and homogeneous cohorts of single categories of mimic diseases. In these investigations, the ALS category itself should be stratified in different disease forms, thus analyzing also most problematic differential diagnostic issues such as the distinction between slowly progressive LMN-predominant ALS forms and neuromuscular diseases exclusively involving LMNs, or between slowly progressive UMN-ALS and PLS.
7) The reason why NFL is more elevated in FTD compared with most other forms of dementia is not yet fully understood (Skillbäck et al., 2014). On one hand, this could simply reflect a more rapid neurodegenerative process as opposed, for example, to what happens in Alzheimer’s disease. On the other hand, a deeper mechanism could be represented by subclinical motor neuron degeneration occurring in a subset of FTD patients harboring TDP-43 pathology (Brettschneider et al., 2014), as suggested by the higher levels of NFL often observed in FTLD-TDP compared with FTLD-tau (Illán-Gala et al., 2021) and by the correlation existing between CSF NFL and the burden of TDP-43 pathology (Olsson et al., 2019).

## Author Contributions

All authors listed have made a substantial, direct and intellectual contribution to the work, and approved it for publication.

## Conflict of Interest

The authors declare that the research was conducted in the absence of any commercial or financial relationships that could be construed as a potential conflict of interest.
